# Supplementary material for: Effects of Lactoferrin on Prevention of Acute Gastrointestinal Symptoms in Winter: A Randomized, Double-Blinded, Placebo-Controlled Trial for Staff of Kindergartens and Nursery Schools in Japan
Source: Int J Environ Res Public Health. 2020 Dec 21;17(24):9582. doi: 10.3390/ijerph17249582 (PMC7767418; doi:10.3390/ijerph17249582)
Supplement: Supplementary file 1 [file ijerph-17-09582-s001.pdf]

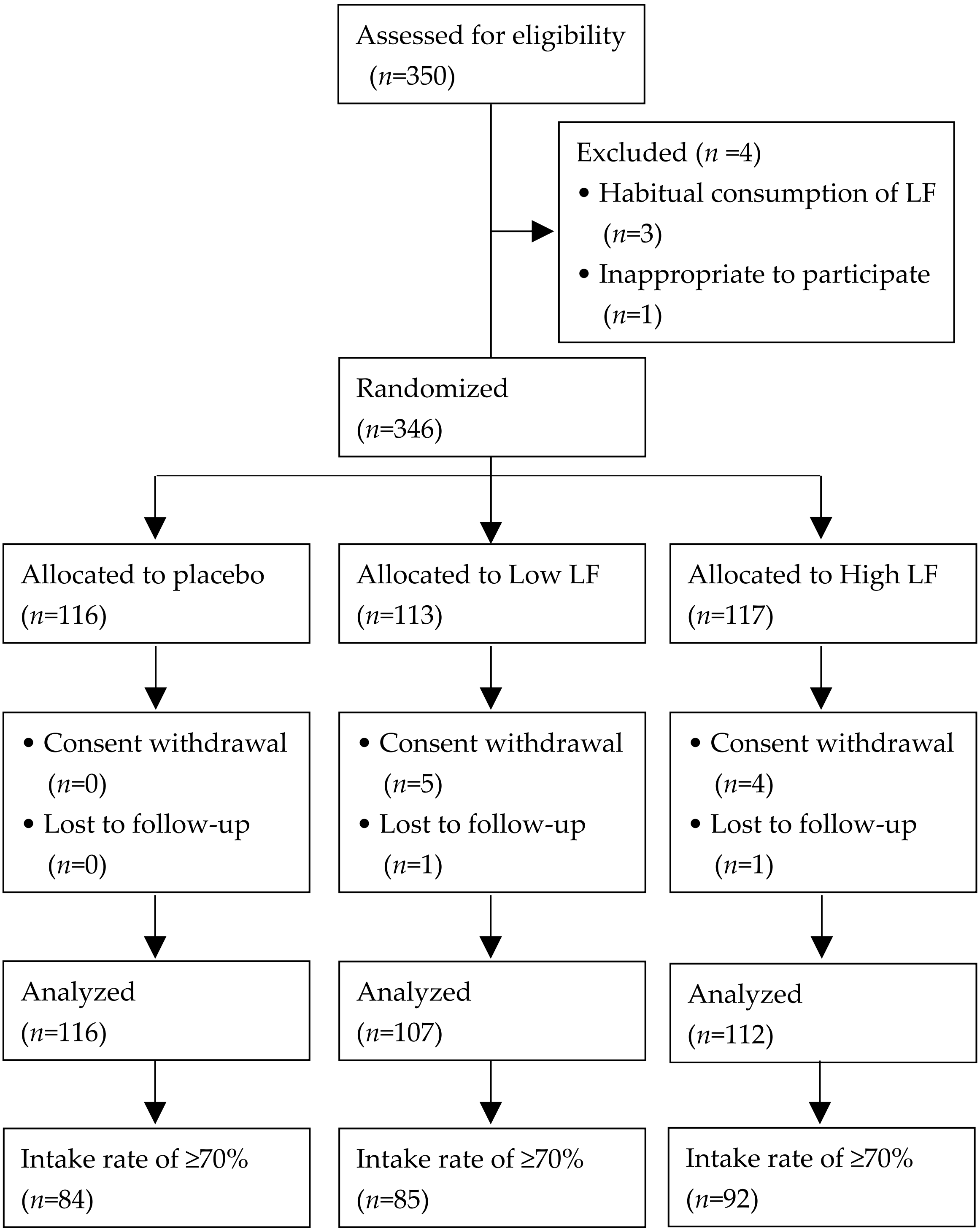


**Figure S1.** CONSORT flow diagram of subjects.

**Table S1**. Cumulative prevalence days of subjective acute gastrointestinal symptoms

| | **Placebo** | **Low LF[1]** | **High LF [2]** | ***p*-Value [1]** | ***p*-Value [2]** | ***p* for trend** |
|---|---|---|---|---|---|---|
| Abdominal pain | | | | | | |
| Positive, day | 50 | 28 | 9 | 0.040 | < 0.001 | < 0.001 |
| Negative, day | 9694 | 8960 | 9399 | | | |
| Nausea | | | | | | |
| Positive, day | 30 | 22 | 9 | 0.488 | 0.001 | 0.002 |
| Negative, day | 9714 | 8966 | 9399 | | | |
| Vomiting | | | | | | |
| Positive, day | 11 | 4 | 6 | 0.123 | 0.333 | 0.236 |
| Negative, day | 9733 | 8984 | 9402 | | | |
| Diarrhea | | | | | | |
| Positive, day | 34 | 17 | 9 | 0.048 | < 0.001 | < 0.001 |
| Negative, day | 9710 | 8971 | 9399 | | | |
| Fever | | | | | | |
| Positive, day | 22 | 8 | 5 | 0.027 | 0.002 | < 0.001 |
| Negative, day | 9722 | 8980 | 9403 | | | |

[1]: placebo vs low LF, [2]: placebo vs high LF.
